# Novel *In-situ* Precipitation Process to Engineer Low Permeability Porous Composite

**DOI:** 10.1038/s41598-018-28786-z

**Published:** 2018-07-16

**Authors:** Swambabu Varanasi, Uthpala Garusinghe, George P Simon, Gil Garnier, Warren Batchelor

**Affiliations:** 1 Bioresource Processing Research Institute of Australia (BioPRIA), Department of Chemical Engineering, Monash University, Clayton, 3800 Australia; 2 Department of Materials Science and Engineering, Monash University, Clayton, 3800 Australia; 3 Indian Institute of Petroleum and Energy (IIPE), Visakhapatnam, India

## Abstract

Inspired by the natural precipitation of minerals in soil and rocks, a novel, simple and industrially scalable *in-situ* precipitation process to produce low permeability porous composites is presented. This process relies on capillary flow in wettable porous composites to absorb and store liquid. In this process, a porous composite first absorbs a salt solution, after which the composite is dipped in a second salt solution. Salts are selected such as they react to form an insoluble precipitate. As big pores absorb more liquid than small pores, the precipitated particles are formed specifically for each pore. In this paper, precipitation of CaCO_3_ nanoparticles in cellulose nanofibre (CNF) films was demonstrated as an example. Precipitation of 1 wt% of CaCO_3_ nanoparticles in the CNF film reduced the pore volume by 50%, without changing the density. This reduced the water vapour and oxygen transmission rates by one order of magnitude to 4.7 g/m^2^.day and 2.7 cc/m^2^.day, respectively. The barrier properties of *in-situ* precipitated composites showed superior performance to previously reported CNF films in literature. The concept is general and of very high industrial interest as it can easily be retrofitted to current continuous industrial processes.

## Introduction

This paper demonstrates a novel and simple method to selectively precipitate inorganic particles of variable and controllable length scale within the porous structure of the material/web in the regions appropriate to greatly reduce the accessible porosity. The specific advantage of this technique is that the precipitated particles will preferentially be located in the pores of the porous material, precisely where it is needed the most to significantly reduce the porosity/permeability of the porous material.

The concept of *in-situ* precipitation is well known for developing materials with interesting properties^1–5^. For example, gold nanoparticles are precipitated on the surface of a glass substrate for architectural applications^1^. Another example is silver nanoparticles are precipitated in a polymer hydrogel to develop biocidal properties^6^. The precipitation of inorganic particles occurs through nucleation growth induced by the super saturation^7^ or mixing^8,9^ of solvent or reagent. In fact, precipitation of mineral in soil or rocks is a natural phenomena^10^. One may easily precipitate particles on the surface of porous web/materials that do not affect the porosity or permeability substantially or one may just clog the pores through precipitation. However, it is difficult to selectively precipitate inorganic nanoparticles in the pore space available over a targeted area^10^. Also, there is no previous literature on the precipitation of nanoparticles in porous webs or materials to selectively close pores, as far as we know. A good deal of work has been done in CaCO_3_ and cellulose composites for applications such as increasing brightness of paper or board, ply board, etc^11–13^. A few precipitation methods have been reported, including the precipitation of CaCO_3_ onto cellulose nanofibres in suspension to coat fibres and reduce their water uptake^3^, or using nanomaterials in suspension to control the pore sizes in which nanoparticles nucleate and grow^5,14–16^. The technique reported in this paper is different in that nanoparticles are nucleated within a preformed web used as absorbing template; the size distribution of nanoparticles matches that of the porous substrate. In this paper, this concept was demonstrated using cellulose nanofibre (CNF) films to control the pore network to improve the oxygen and water vapour barrier performance as the target application. However, this technique is applicable to any hydrophilic porous material/web.

CNFs are fibres of width below 100nm and length of several microns. CNFs are completely derived from renewable resources such as wood, cotton and agricultural wastes^17^. They are currently under investigation for a wide range of applications, including membrane filtration^18,19^, flexible electronics^20^, viscosity modifier for food^21^ and oil drilling fluids^22^, filler and strengthening agents for paper^23^.

An attractive use of CNF materials is as a performance packaging layer, replacing petroleum-derived polymer layers, in polymer-paper laminates to make a new generation of fully renewable, recyclable and biodegradable packaging materials. However, the properties of CNF films must be equal or better than their petroleum-based competitors. While CNF films have greatly superior barrier properties than conventional paper materials, their barrier properties are still deficient compared to those of petroleum based polymers^24^. Two main reasons for poor barrier properties are that the pore size of the CNF films is bigger than the size of most gas/vapour molecules and CNF films are hydrophilic^24^. To mitigate these limitations, nanoparticles or disk type materials such as clay platelets were added to the suspension prior to sheet making, either to reduce the pore size of nanofibre film or to increase tortuosity^25,26^. Although these techniques improve the barrier properties, the random arrangement of these materials in the nanofibre matrix limits the composite performance and also requires large amounts of additive, significantly increasing density and reducing overall porosity.

As another example, a thin layer of CNFs on porous fibrous support is also used in membrane filtration applications. However, it is limited to ultra-filtration application and also molecular weight cut-off of these membranes is high because of high pore size of the skin layer^18,27–29^. Pore size and porosity are the key properties in designing both barrier materials and membranes. Barrier materials need low porosity, while the membranes require high porosity with controlled pore size to ensure good separation^30,31^.

It is the purpose of this study to develop a new, general and industrially scalable method of precipitating inorganic nanoparticles *in situ* within a hydrophilic nanofibre matrix to control the pore structure. In the work here, we have selectively precipitated calcium carbonate from two salts, but the method could also be applied to precursor/reducing agent combinations that are used to precipitate TiO_2_ or Ag nanoparticles. The selective precipitation of small amounts of inorganic nanoparticles within the porous matrix greatly reduces the accessible pore volume. Using cellulose nanofibre barrier layers as example, the effect of reducing accessible pore volume to significantly decrease oxygen and water vapour permeability is investigated. The target is to match or improve the performance of petroleum derived polymer layers.

## Results

This study reports an *in-situ* precipitation method to control the pore size and porosity of wettable porous materials. Here, we demonstrated the technique with precipitating CaCO_3_ nanoparticles from CaCl_2_ and Na_2_CO_3_ salts in CNF films. According to this technique, a CNF film was initially dipped in the CaCl_2_ salt solution (Solution A) until saturated and subsequently in the Na_2_CO_3_ solution (Solution B). Both the salt solutions that were absorbed and retained into the pores of the web or porous material then reacted to precipitate CaCO_3_ particles of variable and controllable length scale within the porous structure of the film. Upon precipitation, the resulting particles or solid precipitates remain contained within the material pores.

**Figure 1:**
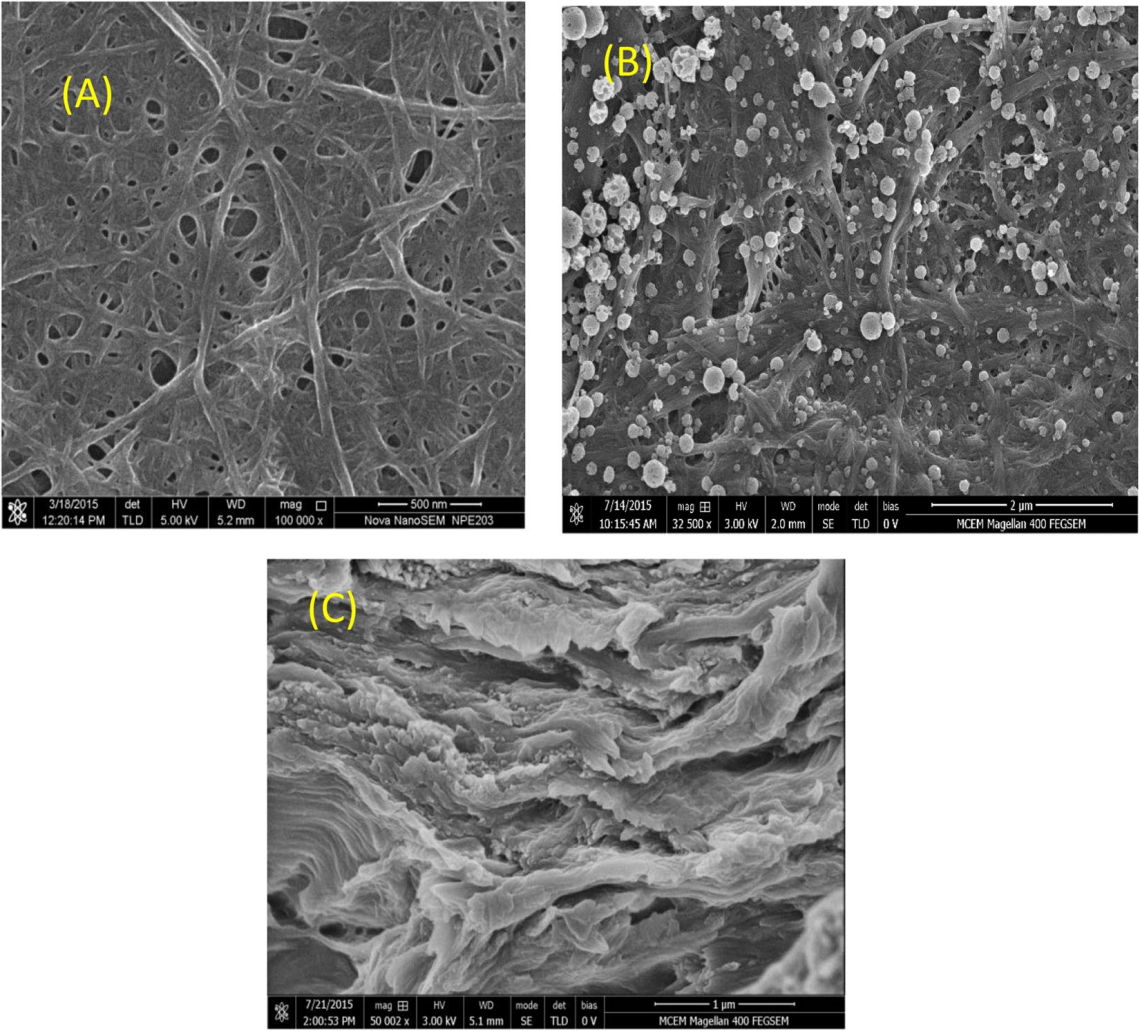
SEM images of CNF film before (**A**) and after (**B** and **C**) *in-situ* precipitation of nanoparticles. Composites treated with 0.2 M solutions – film surface (**B**), cross section (**C**).

Precipitation of CaCO_3_ nanoparticles in the CNF film can be seen in the surface and cross section image of CNF film after precipitation as shown in Fig. 1. Precipitated particles (as seen in Fig. 1B) are in nanoscale range and well distributed spherical structures with smooth surfaces were observed. This phase we denote - following Hostomsky and Jones^32^ and Zou *et al*.^33^- calcite. However, formation of calcite is not direct. Upon dipping the Ca^+ ^^2^ saturated film in solution containing CO^−3^, CO^−3^ ions diffused into film and spontaneously precipitated since we used salt concentrations that are higher than 0.1 M^9,34^. Formed particles are in the nanoscale and spherically shaped particles. The phase of such particles is reported as amorphous calcium carbonate (ACC) in the literature^32,33^. These ACC particles transform into vaterite in the solution. Vaterite is an unstable form. Upon drying, those particles transforms to calcite, the stable crystalline form^33^. A specific advantage of this technique is that particles are precipitated in the pores of the porous material at precisely the location which can most significantly reduce porosity/permeability of the porous film.

**Figure 2:**
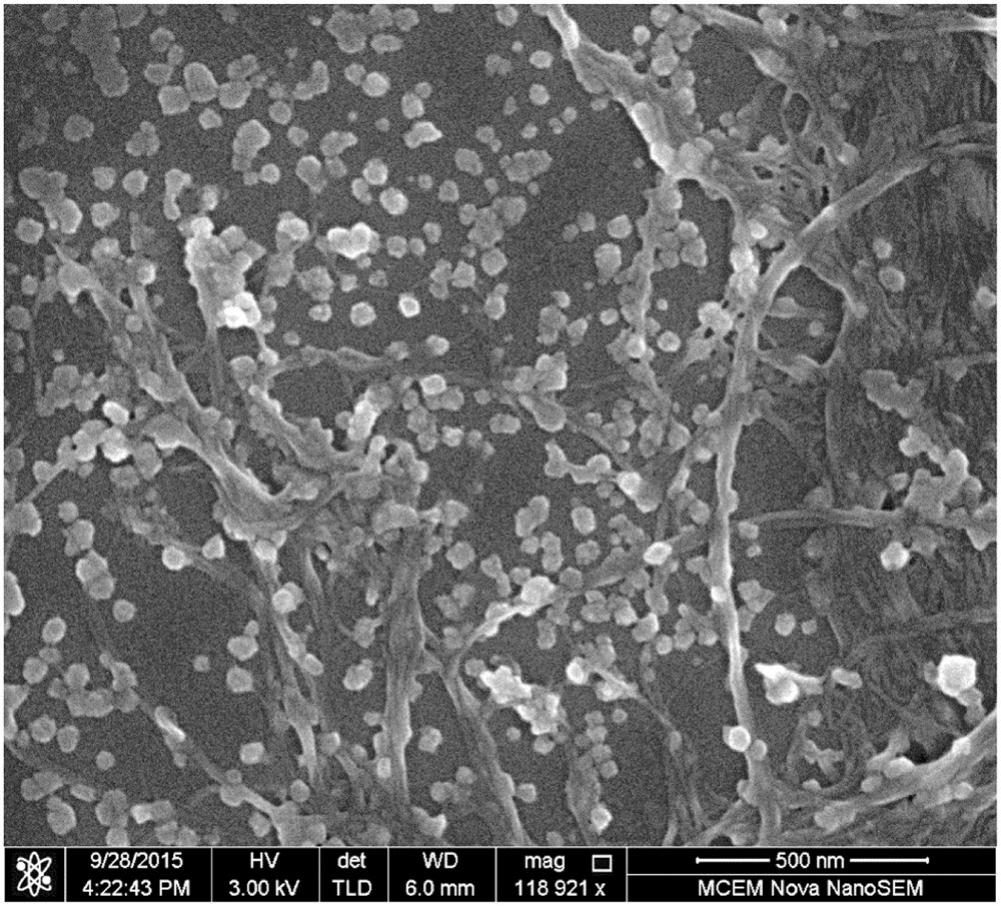
SEM image of *in-situ* precipitated composite suspension after disintegration.

The SEM image of the disintegrated suspension cast on a metal plate is shown in Fig. 2. The appearance of the nanofibres in the disintegrated suspension is similar to the original nanofibre suspension used for preparing composites. Figure 2 also showed that these composites are recyclable, since the fibres and nanoparticles have been fully separated during the hydraulic disintegration process.

**Figure 3:**
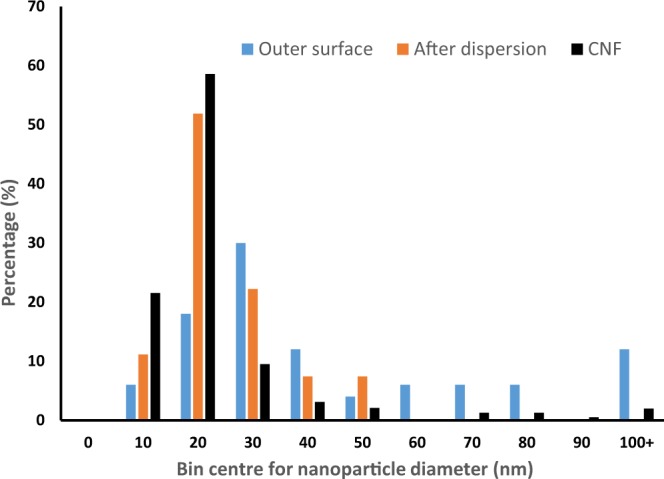
Diameter distribution of CNF and precipitated nanoparticles of composites treated with 0.2 M solutions.

**Figure 4:**
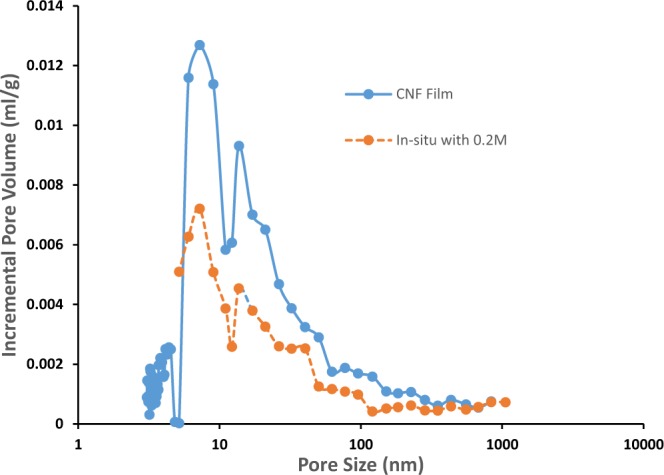
Pore size distribution of CNF film and composite after *in situ* precipitation using 0.2 M solutions.

Figure 3 shows the diameter distributions of the precipitated nanoparticles measured on the surface (from Fig. 1B), from the bulk and surface after disintegrating the composite (from Fig. 2) and of the CNF sample. CaCO_3_ particles precipitated in the absence of the web average 1–2 µm, which is much larger than those precipitated here^35^. Figure 3 shows that the precipitated particles from the bulk and surface are much smaller than the particles on the surface, confirming that the particles seen in Fig. 2 are mostly from the pores of the CNF film. It is interesting to note that the peak in the size distribution of all the nanoparticles is similar to the median diameter of the nanofibres, which was 20 nm. The effect of *in-situ* precipitation in reducing the porosity and controlling the pore size of films can be further understood from the pore size distribution shown in Fig. 4. Two peaks at 7.2 nm and 13.7 nm are visible as well as a broad tail of larger pores. It can be seen that the precipitation has reduced the pore volume by approximately half across the range of pore sizes.

Reduction in permeability was confirmed by the water vapour and oxygen transmission (WVTR and OTR) measurements, which were selected since the barrier performance of a packaging material is typically evaluated based on these properties. When the permeation of water and oxygen is lower i.e, lower permeability, the shelf life of materials such as food or pharmaceuticals in the packaging material is higher. WVTR and OTR of CNF films and composites prepared through *in-situ* precipitation were measured as described in the experimental section and tabulated in Table 1. It can be seen that *in-situ* precipitation greatly reduced the WVTR and OTR of CNF films. The WVTR and OTR of CNF film was 44.7 g/m^2^.day and 20.1 cc/m^2^.day, respectively. Both the WVTR and OTR was further reduced by one order of magnitude with the precipitation of only 1 wt% CaCO_3_ with 0.2 M reactants. Results showed that the precipitation is controlled by the lowest concentration since it is the limiting reactant. Although the precipitation of CaCO_3_ is increased with the higher reactant solution concentration of 0.5 M, WVTR and OTR increased to 10.6 g/m^2^.day and 3.02 cc/m^2^.day.

**Table 1:** Barrier properties of CNF films and composites.

Film	Concentration of Na_2_CO_3_/CaCl_2_ solutions (M)	CaCO_3_ precipitated (wt%)	WVTR (g/m^2^.day) at 23 °C and 50% RH	OTR (cc/m^2^.day) at 23 °C and 50% RH
CNF	0/0	0	44.7	20.1
CNF composite	0.2/0.2	1	4.7	2.7
CNF composite	0.5/0.5	4.2	10.6	3.02
CNF composite	0.2/0.5	1.1	4.9	2.9
CNF composite	0.5/0.2	1.1	5.1	3.01

The mechanical properties of CNF film and composites are given in Table 2. The results showed only a slight reduction in tensile strength and modulus. This may be due to the rewetting of film during *in-situ* precipitation. Interestingly, it appears that precipitation of CaCO_3_ did not greatly affect the bonding between the nanofibers, as evidenced by similar tensile strength values.

**Table 2:** Mechanical properties of CNF film and composites with 95% confidence intervals in brackets.

Nanofibre film	Concentration of Na_2_CO_3_/CaCl_2_ solutions (M)	Tensile Strength (MPa)	Modulus (GPa)
CNF film	0	96.9 (25.68)	5.39 (0.49)
CNF composite	0.2/0.2	91.96 (14.99)	4.59
CNF composite	0.5/0.5	86.95 (26.46)	4.52

## Discussion

*In-situ* precipitation selectively closed the surface and some of the internal pores with CaCO_3_ nanoparticles. Hence, the pore size and porosity of composite are significantly decreased. This can be clearly understood from the pore size distribution results given in Fig. 4, which show that the selective precipitation of only 1 wt% of CaCO_3_ nanoparticles has reduced the pore volume measured with mercury porosimetry by approximately half. It is interesting to note that this reduction in pore volume occurs uniformly across the full measured range of pore sizes.

This reduction in pore volume is not mainly due to the precipitation of CaCO_3_ particles filling these pores. If we consider 1 m^2^ of a 60 g/m^2^ nanofibre sheet, with a measured thickness of 70 µm, the total pore volume available in the sheet, assuming a cellulose density of 1500 kg/m^3^ is 3.0 × 10^−5^ m^3^ and completely filling this with precipitated CaCO_3_ would have produced a mass of 81 g/m^2^, given a CaCO_3_ density of 2710 kg/m^3^. Given that the molar masses of Na_2_CO_3_ and CaCl_2_ are 106 and 111 g/mol, respectively, then the theoretical maximum precipitated mass of CaCO_3_, assuming sequential saturation of the two liquids is 0.6 g/m^2^ for 0.2 M solutions, matching the measured precipitated mass of 0.6 g/m^2^ of sheet. This mass however will fill only 0.7% of the available pore space.

The 50% reduction in pore volume measured by mercury porosimetry, given that only 0.7% of the estimated pore space is filled, must be due to the precipitates blocking paths within the porous structure, rendering large sections of the pore structure inaccessible to mercury intrusion. This is a remarkable result. We have made large sections of the pore structure inaccessible, without significantly changing the density. This large reduction in accessible pore volume has in turn led to the reduction in water vapour and oxygen permeability.

Although the mass of precipitated CaCO_3_ increased with the higher reactant solution concentration of 0.5 M, WVTR and OTR also increased to 10.6 g/m^2^.day and 3.0 cc/m^2^.day. This is due to the increase in concentration of the reactants increasing the precipitation of NaCl on the film, which increases the absorption and diffusion of water vapour^36^.

In order to compare the barrier properties of CNF films and composites reported in this paper with literature data, water vapour transmission rate (WVTR) (g/m^2^.day) and oxygen transmission rate (OTR) were normalized to permeability using equations (1) and (2), respectively, as given in the ASTM standard method. All the values are tabulated in Table 3.

1 $${\rm{Water}}\,{\rm{Vapour}}\,{\rm{Permeability}}\,(\mathrm{WVP})=\frac{WVTR\ast t}{S(R{H}_{1}-R{H}_{2})}$$

2 $${\rm{Oxygen}}\,{\rm{Permeability}}\,({\rm{OP}})=\frac{OTR\ast t}{p{o}_{2}}$$

*S* = Saturation vapour pressure at test temperature in atm; *RH*_1_ = Relative humidity of the test chamber expressed as a fraction; *RH*_2_ = Relative humidity of the vapour sink expressed as a fraction; *t* = Thickness of film or composite in microns; *po*_2_ = partial pressure of oxygen in Kpa.

**Table 3:** WVP and OP of CNF films and composites and polymeric films.

Material	Water Vapour Permeability (WVP) (g.mm/m^2^.day.atm) 23 °C and 50% RH	Oxygen Permeability (OP) (cc.µm/m^2^.kPa.day) 23 °C and 50% RH
CNF Film (in this paper)	193	11.8
CNF composite (in this paper)	20.3	1.6
CNF film literature	707^38,39^	3.52
Acetylated CNF films	553^39^	—
Carboxymethylated CNF film	—	0.85^38,40^
Polyethylene terephthalate (PET)	13^41^	1–5^41^
Ethylene Vinyl Alcohol (EVOH)	8.8^42^	0.01^41^
Low density polyethylene (LDPE)	1.6^42^	3353^42^
Polypropylene (PP)	2.16^41^	50–100^41^
Poly Styrene (PS)	20.2	100–150^41^

WVTR from literature of a number of fossil fuel derived polymers used in packaging are also converted to permeability as per equation 1 and compared and measured at the same conditions of 23 °C and 50% RH.

Results show that WVP and OP of CNF composites reported in this paper are much lower than values of CNF films reported elsewhere in literature, and demonstrate similar performance to that of either PS or PET films. OP of carboxymethylated CNF film is lower than CNF composite. Although the WVP of our CNF composites are higher than LDPE and PP films, the OP of the CNF composites is much lower. Only the EVOH showed superior barrier properties to CNF composites in both WVP and OP. The barrier properties of the *in-situ* precipitated composites showed superior performance to LDPE, PS, PP and PET in one of WVP or OP.

## Conclusion

A novel and simple diffusion induced *in-situ* precipitation method was developed to selectively precipitate CaCO_3_ particles within the porous structure of cellulose nanofibre film. This process relies on capillary flow in wettable porous composites to absorb liquid. A porous composite first absorbs a salt solution, after which the composite is dipped in a second salt solution. Salts are selected to react and form an insoluble precipitate. As big pores absorb more liquid than small pores, the precipitated particles are formed specifically for each pore, providing unique filling capability, and drastically reduce porosity where and as it is needed.

The value of *in-situ* precipitation of CaCO_3_ nanoparticles in cellulose nanofiber films was demonstrated to reduce Oxygen and water vapour permeability for packaging application. The selective precipitation of only 1wt% of CaCO_3_ nanoparticles has reduced the pore volume of CNF film half without significantly changing the composite density. This is due to the precipitates blocking paths within the porous structure, rendering large sections of the pore structure inaccessible to oxygen and moisture permeation. This large reduction in accessible pore volume reduced the water vapour and oxygen permeability by one order of magnitude without deteriorating the mechanical strength of the film. Oxygen permeability of CNF composites is lower than most polymeric films at the exception of EVOH. CNF composites showed competitive water permeability with many common polymers used in packaging. Precipitation method is general and could be applied to pore control in any other wettable porous composites. This process opens new horizons for producing low cost packaging materials and separation membranes where renewability, recyclability and biodegradability are important.

## Experimental

### Materials

Microfibrillated cellulose (MFC) supplied from Daicel Chemical Ltd (Celish KY-100G grade) with 25 wt% solids concentration was used as received as a feed stock to prepare cellulose nanofibers (CNF). Sodium Carbonate (Na_2_CO_3_) and Calcium Chloride (CaCl_2_) were purchased from Sigma Aldrich, Australia.

### Methods

#### Cellulose nanofibers (CNF) preparation

To make CNF, MFC suspension was diluted from 25 wt% to 0.5 wt% solids concentration and the agitated in a 3L Mavis Engineering (Model No. 8522) disintegrator for 15,000 revolutions. The MFC fibre suspension was then homogenized at 1000 bar pressure using a GEA high pressure homogenizer (PANDA 2000) for 5 passes.

#### Diameter distribution

CNF sample diameter distribution was measured from Scanning Electron Microscopy (SEM) images, taken using secondary electron mode with a Nova Nano SEM at a voltage of 5 kV, of a drop of suspension that was cast on a metal plate, air dried and then platinum coated. From each image, the width of each nanofibre observable in the image was manually measured using ImageJ software. The fibre diameters measured in each image were then sorted into bins of 10 nm size and normalized to % percentage.

#### CNF film preparation

CNF films with 60 g/m^2^ basis weight were prepared using a filtration method, as we have reported earlier in^37^. In brief, CNF suspension with 0.3 wt% solids concentration was poured into a chamber having a filter mesh with pore size of 125 μm and then filtered with 5 MPa vacuum. With the help of blotting paper, the wet film was separated from the filter mesh and then dried using automatic sheet drier at a temperature of 120 °C.

#### CNF composite preparation through *in-situ* precipitation

*In-situ* precipitation of CaCO_3_ nanoparticles in the CNF film was carried with Na_2_CO_3_ and CaCl_2_ solutions using the following reaction:

$${{\rm{Na}}}_{2}{{\rm{CO}}}_{3}\,(\mathrm{aq})+{{\rm{CaCl}}}_{2}\,({\rm{aq}})\to {{\rm{CaCO}}}_{3}\,(\mathrm{solid})+2{\rm{NaCl}}\,(\mathrm{aq})$$

Initially Na_2_CO_3_ and CaCl_2_ solutions with the same molarity, such as 0.2 M or 0.5 M, were used. First, the CNF film was dipped in Na_2_CO_3_ solution until it was saturated and then the CNF film was dipped in CaCl_2_ solution until nanoparticles precipitated in the film. The composite was then washed thoroughly with water to remove aqueous NaCl and any excess precipitated CaCO_3_. The sheet was then dried with a hotplate at 140 °C temperature. Some residual NaCl may remain in the structure. Later, Na_2_CO_3_ and CaCl_2_ solutions with different molarity, for example Na_2_CO_3_ solution with 0.5 M and CaCl_2_ solution with 0.2 M, were also used to prepare the composite. All solutions were partially saturated, with concentrations well under the saturation concentration of 96 and 745 g/l for Na_2_CO_3_ and CaCl_2_, at 20 °C, respectively.

#### Precipitated nanoparticles diameter distribution

CNF composite surfaces and cross sections were platinum coated prior to imaging using secondary electron mode with a Magellan FEGSEM at a voltage of 5 kV. Samples were freeze fractured with liquid nitrogen for imaging cross sections of the composites. From each image, diameters of observed nanoparticles in the images were measured using Image J software. The particle diameters measured in each image were then sorted into bins of 10 nm size and normalized to % percentage.

To clearly observe the precipitated particles, CNF composites were disintegrated with a 3L Mavis Engineering (Model No. 8522) disintegrator for 75,000 revolutions after soaking the composite in water overnight. A drop of disintegrated suspension was cast on a metal plate, air dried, platinum coated and then imaged using secondary electron mode with a Nova Nano SEM at voltage of 3 kV.

#### Measurement of the amount of CaCO_3_ precipitated

Ash testing was conducted by incinerating the composites at a temperature of 600 °C according to the standard method TAPPI T211, in order to measure the amount of CaCO_3_ precipitated in the composites.

#### Barrier properties measurement

The water vapour transmission rate (WVTR) and oxygen transmission rate (OTR), were measured using MOCON PERMATRAN 3/31 model and OX-TRAN machines, respectively, at 23 °C and 50% RH. Films were dried at 105 °C for 4 hours prior to testing. OTR testing is made in accordance with ASTM D-3985 and ASTM F-1927 methods. WVTR testing is made in accordance with ASTM F1249 and TAPPI T557 methods.

#### Pore size distribution measurements

Pore size distribution of CNF films and composites were measured using mercury porosimetry (Micromeritics’ AutoPore IV 9500 Series). The films were cut into 4 mm by 4 mm pieces, placed in the sample holder, and then degassed overnight at 105 °C. Samples were then transferred into a penetrometer (0.412 stem, solid) and analysed. The minimum size of pore that can be measured using mercury porosimetry is 3 nm.

#### Mechanical properties

The thickness and tensile strength of films were measured according to Australian/New Zealand Standard Methods 426 s and 437 s, respectively. Films were conditioned for 24 hours at 23 °C temperature and 50% RH prior to testing the mechanical properties.
